# Characterization of two novel intronic OPA1 mutations resulting in aberrant pre-mRNA splicing

**DOI:** 10.1186/s12881-017-0383-x

**Published:** 2017-02-28

**Authors:** Ramona Bolognini, Christina Gerth-Kahlert, Mathias Abegg, Deborah Bartholdi, Nicolas Mathis, Veit Sturm, Sabina Gallati, André Schaller

**Affiliations:** 1 Division of Human Genetics, Department of Pediatrics, Inselspital, Bern University Hospital, University of Bern, Freiburgstrasse 15, 3010 Bern, Switzerland; 2 Department of Ophthalmology, University Hospital Zurich, Zurich, Switzerland; 3 Department of Ophthalmology, Cantonal Hospital St. Gallen, St. Gallen, Switzerland; 4 Department of Ophthalmology, Inselspital, Bern University Hospital, University of Bern, Bern, Switzerland; 5 Graduate School for Cellular and Biomedical Sciences (GCB), University of Bern, Bern, Switzerland

**Keywords:** OPA1, Splice site mutation, Autosomal dominant optic atrophy, Kjer type optic atrophy, Optic neuropathies

## Abstract

**Background:**

We report two novel splice region mutations in *OPA1* in two unrelated families presenting with autosomal-dominant optic atrophy type 1 (ADOA1) (ADOA or Kjer type optic atrophy). Mutations in *OPA1* encoding a mitochondrial inner membrane protein are a major cause of ADOA.

**Methods:**

We analyzed two unrelated families including four affected individuals clinically suspicious of ADOA. Standard ocular examinations were performed in affected individuals of both families. All coding exons, as well as exon-intron boundaries of the *OPA1* gene were sequenced. In addition, multiplex ligation-dependent probe amplification (MLPA) was performed to uncover copy number variations in *OPA1*. mRNA processing was monitored using RT-PCR and subsequent cDNA analysis.

**Results:**

We report two novel splice region mutations in *OPA1* in two unrelated individuals and their affected relatives, which were previously not described in the literature. In one family the heterozygous insertion and deletion c.[611-37_611-38insACTGGAGAATGTAAAGGGCTTT;611-6_611-16delCATATTTATCT] was found in all investigated family members leading to the activation of an intronic cryptic splice site. In the second family sequencing of *OPA1* disclosed a *de novo* heterozygous deletion c.2012+4_2012+7delAGTA resulting in exon 18 and 19 skipping, which was not detected in healthy family members.

**Conclusion:**

We identified two novel intronic mutations in *OPA1* affecting the correct *OPA1* pre-mRNA splicing, which was confirmed by *OPA1* cDNA analysis. This study shows the importance of transcript analysis to determine the consequences of unclear intronic mutations in *OPA1* in proximity to the intron-exon boundaries.

**Electronic supplementary material:**

The online version of this article (doi:10.1186/s12881-017-0383-x) contains supplementary material, which is available to authorized users.

## Background

Autosomal dominant optic atrophy type 1 (ADOA1, MIM#165500), also known as Kjer-type optic atrophy [1, 2], is the most frequent form of inherited optic neuropathies (ION), with a prevalence ranging from 1:10,000 [3, 4] in Denmark to 1:50,000–30,000 [3–6] worldwide. ADOA is typically characterized by childhood-onset insidious moderate to severe progressive bilateral visual loss, color vision deficits, centrocecal scotoma, temporal optic nerve pallor and macular ganglion cell loss [7], which often leads to legal blindness [8–10]. Although ADOA is genetically heterogeneous [11–13], the vast majority of ADOA patients (approx. 75%) harbor a mutation in the *OPA1* gene, less frequent mutations in *OPA3* and *WFS1* are observed in ADOA patients. *OPA1* codes for a mitochondrial dynamin-like GTPase [14–16], which is an inner membrane protein targeted to mitochondria by a highly basic N-terminal import sequence and is anchored to mitochondrial cristae where it faces the intermembrane space [17]. *OPA1* mRNA is 6492 bp (largest isoform) in length and has been mapped to chromosome 3q28-q29 [15, 18, 19]. *OPA1* consists of 31 exons of which the last one is non-coding [15, 19]. Through alternative splicing of exon 4, 4b and 5b, eight different mRNA isoforms with different expression patterns in different tissues can be produced [20]. The OPA1 protein of the main isoform counts 960 amino acids and contains 5 different domains, the N-terminal hydrophobic mitochondrial targeting domain, a N-terminal coiled-coil domain, a GTPase domain, a dynamin central region and a proposed GTPase effector domain and the C-terminal coiled-coil domain [14, 21]. *OPA1* is ubiquitously expressed in human tissues with the highest levels found in retinal ganglion cells and the brain [20, 21]. OPA1 is involved in many different processes. It is the key player during mitochondrial inner membrane fusion, it plays a role in mitochondrial DNA, respiratory chain and membrane potential maintenance, cristae organization by controlling their shape and structure and it is further involved in cytochrome c mediated apoptosis [22–25]. More than 340 unique DNA variants have been allocated to *OPA1* (http://mitodyn.org/home.php?select_db=OPA1) [26]. Half of these mutations result in haploinsufficiency of *OPA1* by premature termination of translation leading to a truncated protein. Most of these mutations are located in the GTPase domain, whereas no mutation was found in the alternatively spliced exons (4, 4b and 5b). The phenotypic expression of *OPA1* mutations in ADOA shows a high variability between families as well as members of the same family [27], often with an incomplete penetrance ranging from asymptomatic carriers and mild visual impairment to legally blind individuals [28]. ADOA can be classified either as a non-syndromic or isolated form, presenting with pure ophthalmologic features or as syndromic form associated with extra-ocular neurological manifestations, known as ADOA plus syndrome [5, 29]. Around 20% of ADOA patients develop additional neuro-muscular features, often in the third decade of life, which are sometimes present at a subclinical level. These include sensorineural deafness, ataxia, axonal sensory-motor polyneuropathy, mitochondrial myopathy and external ophthalmoplegia, whereas in one case parkinsonism and dementia were reported [30]. Many different mutations have been identified in *OPA1* responsible for ADOA, including nonsense mutations, missense mutations, frameshift mutations, in-frame deletions and splice site or splice region mutations [14, 28, 31]. Since the vast majority of *OPA1* mutations are predicted to result in a premature stop codon, mainly caused by splice site or splice region, nonsense or frameshift mutations, the generation of a truncated protein leading to haploinsufficiency is proposed to be the major cause for the pathogenesis of ADOA [28]. The majority of transcripts containing a premature termination codon (PTC) are degraded by a mechanism called nonsense-mediated decay (NMD), which is used to protect the cell from translation of potentially harmful proteins [32]. In this study we analyzed all coding exons including the intron-exon boundaries of *OPA1* by DNA sequencing of two unrelated families presenting with clinical features of ADOA. We report two novel splice region mutations in *OPA1,* which we characterized at RNA level, thereby expanding the mutational spectrum of *OPA1.*

## Methods

### Patients

#### Family 1

The index patient (III:1, Fig. 1a) was referred to our institutions at age of 9 years because of reduced vision and optic disc pallor. The child is the first child of unrelated parents of Swiss origin. She is normally developed and besides her vision disorder a healthy child. On the first examination, best corrected visual acuity (BCVA) for distance was reduced to 0.3 and 0.5, which dropped to 0.2 and 0.32 at 10 years in the right and left eye, respectively. Temporal optic atrophy was present in both eyes. The apparently unaffected mother (II:2) showed minor optic atrophy, which was confirmed by the reduced retinal nerve fiber layer thickness in the papillo-macular bundle (Fig. 2a). BCVA was normal in the left eye but reduced to 0.8 in the right eye due to anisometropic amblyopia (Fig. 2a). The maternal grandmother (I:2) was already diagnosed with optic atrophy with mild (age 55: BCVA 0.7 in both eyes) but progressive vision loss (age 62: BCVA 0.4 and 0.2).

**Fig. 1:**
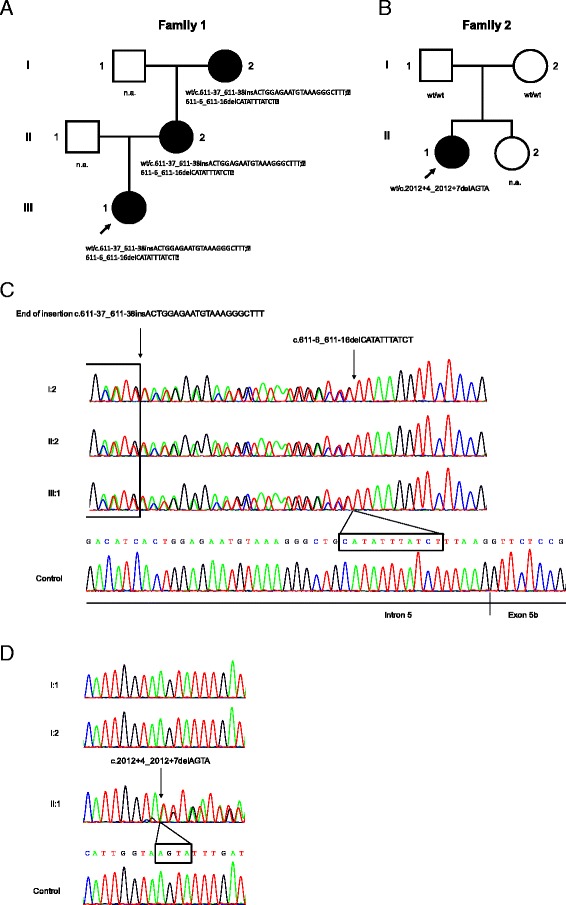
**a** and **b** Pedigrees of the described and analyzed ADOA families. Index patients are indicated with an arrow. Black symbols represent affected individuals. Symbols containing ‘n.a.’ (not analyzed) indicate individuals with unknown genotype and/or phenotype (not described in this study). **c** Sequence analysis of the *OPA1* gene at the genomic level. All affected family members of family 1 carrying a heterozygous intronic 22 bp insertion c.611-37_611-38insACTGGAGAATGTAAAGGGCTTT and a 11 bp deletion c.611-6_611-16delCATATTTATCT (indicated with the arrow). **d** Sequence analysis of *OPA1* in the index patient of family 2 discloses a heterozygous intronic 4 bp deletion c.2012+4_2012+7AGTA which was absent in healthy parents and the control

**Fig. 2:**
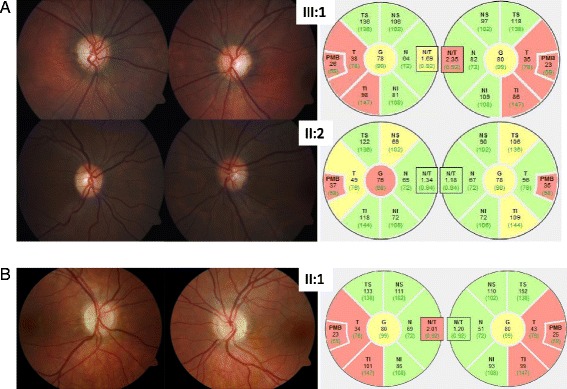
**a** Color fundus photography depicting optic disc atrophy of the temporal area is more pronounced in the index patient III:1 of family 1 than in her mother II:2. Corresponding retinal nerve fiber layer thickness analysis by optical coherence tomography demonstrating slight (*yellow sectors*) to significant reduction (*red*) in particular within the papillomacular bundle (PMB). **b** Ophthalmoscopic examination of the index patient II:1 of family 2 demonstrates bilateral temporal optic disc pallor and peripapillary optical coherence tomography demonstrates a thinning of the retinal nerve fibre layer most pronounced in the papillomacular bundle

The diagnosis of ADOA was established and molecular genetic testing performed.

#### Family 2

The index patient (II:1, Fig. 1b) is the first child of unrelated parents of Swiss and Italian origin. The parents (I:1 and I:2) and her younger sister (II:2) did not complain of any visual dysfunction. There is a possible history of a mitochondrial disorder with myopathy and ptosis in the paternal family (paternal grandmother), but no genetic testing had been performed with regard to this suspicion.

Personal history: The patient was born after an uneventful pregnancy and psychomotor development was normal. At age 14 years she noticed a decrease of visual acuity at distance. This led to the diagnosis of a myopia of -1.0 diopters in both eyes and glasses were prescribed. However, despite best correction, visual acuity remained at 0.63 in both eyes. Visual field showed few unspecific defects in the central 30°, mean visual field defect was 1.1 dB and 1.5 dB in the right and the left eye respectively. The patient did not notice difficulties with color vision and she correctly identified 14 and 13 out of 15 Ishihara color plates with the right eye and the left eye, respectively. Ophthalmoscopic examination demonstrated bilateral temporal optic disc pallor (Fig. 2b) and peripapillary optical coherence tomography showed a thinning of the retinal nerve fibre layer most pronounced in the papillomacular bundle (Fig. 2b). Visual evoked potentials were not recorded and brain MRI scan ruled out a compression of the optic nerves. There was no hearing impairment but diffuse myalgia, which became manifest about the same time as the decrease in visual acuity. A thorough neurological exam did not reveal any abnormal findings apart from the visual impairment. Therefore, the tentative diagnosis of ADOA was made at age 14 years and genetic analysis of the *OPA1* gene was initiated.

### Molecular genetic studies

#### Mutation screening and DNA Sequencing

Genomic DNA (gDNA) was isolated from peripheral white blood cells of EDTA-stabilized venous blood, using Prepito DNA Blood600 kit according to the manufacturer’s instructions (PerkinElmer, Waltham, MA, USA). All coding exons (including exon 4b and 5b) of *OPA1* as well as exon-intron boundaries were amplified from gDNA by PCR (primers and cycling conditions are listed in Additional file 1: Table S1). All PCR reactions were performed in a Veriti thermal cycler (Thermo Fisher, Waltham, MA, USA) and fragments were analyzed using Qiaxcel DNA High Resolution Cartridge (Qiagen GmbH, Hilden, Germany). PCR products were ExoSAP (Thermo Fisher, Waltham, MA, USA) purified and sequenced using BigDye Terminator chemistry v3.1 (Applied Biosystems, Foster City, CA, USA). Sequencing of both strands was performed on an ABI Prism 3500XL Genetic Analyzer (Applied Biosystems, Foster City, CA, USA). Sequence data were analyzed with SeqAnalysis 6 and SeqScape 3 (Applied Biosystems, Foster City, CA, USA). MLPA reactions were performed using the P229 kit from MRC Holland (Amsterdam, Netherlands) according to the manufacturer’s instructions. Products of the MLPA reaction were separated on an ABI Prism 3500XL Genetic Analyzer (Applied Biosystems, Foster City, CA, USA) and were analyzed using GeneMarker Software v2.6.3. (SoftGenetics, State College, PA, USA)

GenBank transcript NM_130837 was used for variant numbering using A in ATG as number 1.

#### OPA1 cDNA analysis

Total RNA was obtained from PAX-blood (family 1, family 2 and a healthy unrelated control subject) using the PAXgene Blood RNA kit according to the manufacturer’s recommendations (PreAnalytiX GmbH, Hombrechtikon, Switzerland). Random oligohexamer primed RNA (up to 1 μg) was reverse-transcribed using the SuperScript II First-Strand Synthesis System (Invitrogen, Carlsbad, CA, USA) in a total volume of 25 μl, according to the manufacturer’s protocol. 1 μl of the single-stranded cDNA was used as template for amplification of fragments extending from *OPA1* exon 4b to 5b (243 bp) and exon 17 to 23 (605 bp), using the following primer pairs: OPA1-Ex4bF (5’-CTCAGGTCACAAATTGGTTAGT-3’), OPA1-Ex5bR (5’-CAACAGAAGCGCAAGGTGTC-3’), and OPA1-Ex17F (5’-TCAAAGCTCCTAAAGACAAGC-3’) and OPA1-Ex23R (5’-AGCTTGAGGGTTATTCAACA-3’). PCR conditions using HotStar Taq DNA Polymerase (Qiagen GmbH, Hilden, Germany) included an initial denaturation step of 15 min at 95 °C, 40 cycles of denaturation at 94 °C for 30 s, annealing at 55 °C for 30 s, polymerization at 72 °C for 1 min and a final extension step at 72 °C for 10 min RT-PCR products were separated on a 12% polyacrylamide gel and extracted overnight in dH_2_O at 4 °C. Isolated RT-PCR products were re-amplified using the same cycling conditions and primers as described above, except of 32 amplification cycles instead of 40. Re-amplified PCR products were used for subsequent sequencing as described above.

## Results

### Mutation analysis in OPA1

All coding exons of *OPA1*, including exon-intron boundaries, were sequenced and analyzed in two ADOA patients from two unrelated families (family 1: III:1 and family 2: II:1) and their family members (Fig. 1a and b). Sequence analysis of patient 1 (III:1, family 1) revealed a heterozygous 22 bp insertion c.611-37_611-38insACTGGAGAATGTAAAGGGCTTT proximal of a 11 bp deletion c.611-6_611-16delCATATTTATCT in intron 5 (Fig. 1c), whereas the insertion c.611-37_611-38insACTGGAGAATGTAAAGGGCTTT could be a potential result of a tandem duplication (Fig. 4). This sequence alteration is likely to abolish the 3’ acceptor splice site located in intron 5, which was supported by different in silico prediction tools (Table 1). The patient’s mother presents with mild visual impairment and optic atrophy (II:2, family 1) and a grandmother with reduced vision and optic atrophy (I:2, family 1). Both of them were found to harbor the same heterozygous c.[611-37_611-38insACTGGAGAATGTAAAGGGCTTT;611-6_611-16delCATATTTATCT] mutation (Fig. 1c).

**Table 1:** In silico prediction of splice score changes of the natural splice site

		Splicing prediction	
Prediction Tool	Threshold	Acceptor Site	Donor Site
		c.611-16 _611-6del c.611-37_﻿611-38ins	c.2012 + 4_2012 + 7del
SSF	≥70	87.40 ⇒ —	87.36 ⇒ —
MaxEnt	≥0	7.78 ⇒ —	10.47 ⇒ 2.39 (-77.2%)
NNSPLICE	≥0.4	0.93 ⇒ —	1.00 ⇒ —
GeneSplicer	≥0	n.a.	2.26 ⇒ —

*n.a*. not analysed, *SSF* SpliceSiteFinder-like [39], *MaxEnt* MaxEntScan [40], *NNSPLICE* Splice Site Prediction by Neural Network [41], GeneSplicer [42]; Threshold: minimal value to recognise a putative splice site; - splice site abolished

In index patient 2 (II:1, family 2) we identified a heterozygous 4 bp deletion c.2012 + 4_2012 + 7delAGTA in intron 19 (Fig. 1d), which was reported once in a patient suspicious for ADOA (http://www.mitodyn.org/variants.php?select_db=OPA1&action=view&view=0000543%2C0000353%2C0) and classified as variant of unknown clinical significance (VOUS). This mutation was predicted to abolish the 5’ splice donor site (Table 1). We analyzed further both unaffected parents (I:1 and I:2, family 2), who were negative for the heterozygous c.2012 + 4_2012 + 7delAGTA deletion (Fig. 1d), suggesting the mutation occurred *de novo* in the index patient. MLPA analysis did not give any evidence for the presence of the detected intronic mutations. Furthermore, in both index patients, the three LHON mutations m.11778G > A, p.(Arg340His) in *MTND4*, m.3460G > A, p.(Ala52Thr) in *MTND1* and m.14484 T > C, p.(Met64Val) in *MTND6* were not detected. Also no mutations were found in the nuclear genes *OPA3*, *TMEM126A* and *WFS1*, thus no other reason for an opticusatrophy could be identified.

### OPA1 cDNA analysis

**Fig. 3:**
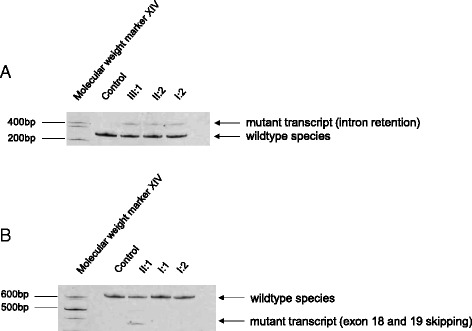
Transcript analysis of family 1 (**a**) and family 2 (**b**). **a** All affected relatives of the index patient (III:1) show an additional transcript species compared to the control. **b** Index patient (II:1) has an additional transcript compared to the control and the healthy parents

**Fig. 4:**
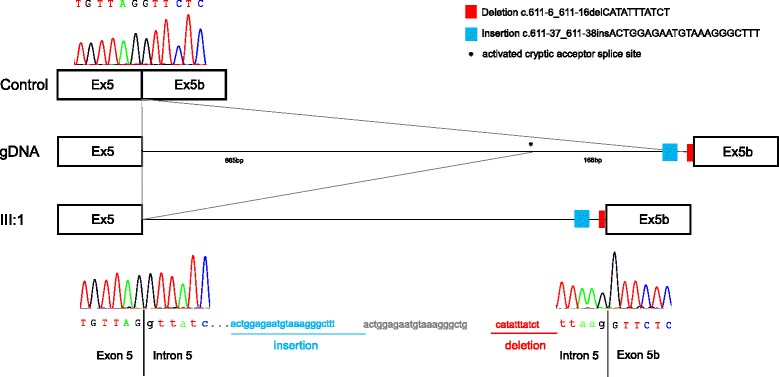
Sequence analysis of *OPA1* transcripts. Sequencing of an additional larger transcript species found in the index patient (III:1) identified an activation of a cryptic acceptor splice site leading to the retention of intronic sequence containing an insertion of 22 bp c.611-37_611-38ACTGGAGAATGTAAAGGGCTTT (blue boxes) flanked by intronic sequence (*light gray*). Red boxes represent the deletion c.611-6_611-16CATATTTATCT. Intronic sequence is written with small letters and exonic sequence with capital letters. (*) indicates the activation of the cryptic splice site, which is located in the wildtype sequence, 166 bp upstream of the canonical splice site. This transcript species was also identified and sequenced in the affected family members (I:2 and II:2) (sequence data not shown)

Both identified deletions have been predicted to alter the splice sites using different *in silico* prediction tools (Table 1). Since *OPA1* is expressed in many tissues, including the white blood cells [21, 28], we investigate the impact of the deletions at RNA level using PAX-blood samples of various family members (family 1 (I:2, II:2 and III:1) and family 2 (I:1, I:2 and II:1)). cDNA analysis in family 1, showed two transcript species in all investigated and affected family members (family 1: index patient III.1, mother II:2 and grandmother I:2), including the wildtype form and an additional larger transcript (Fig. 3a). Sequence analysis of the transcripts showed that the c.[611-37_611-38insACTGGAGAATGTAAAGGGCTTT;611-6_611-16delCATATTTATCT] results in the activation of a cryptic acceptor splice site, which is located in the wildtype sequence 166 bp upstream of the canonical 3’ splice acceptor site. Activation of this cryptic splice site leads to an addition of 177 intronic bases in the aberrantly spliced mRNA inserting a premature stop codon in the context of the appropriate reading frame of this transcript, including the 22 bp insertion c.611-37_611-38insACTGGAGAATGTAAAGGGCTTT (Fig. 4), which is also encoded by the gDNA (Fig. 1c).

**Fig. 5:**
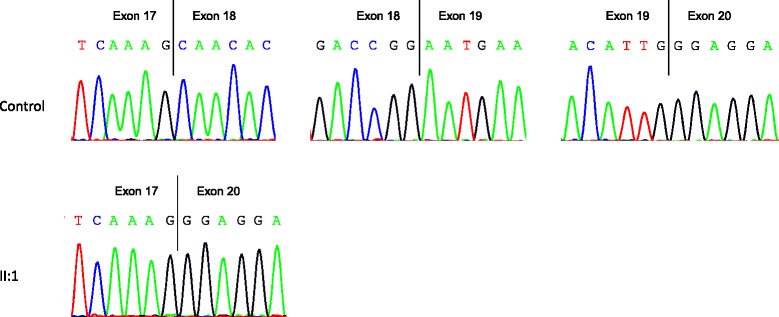
Sequence analysis of *OPA1* transcripts. Sequencing of the additional smaller transcript species in the index patient (II:1) disclose a skipping of exon 18 and 19. This transcript species was not found in the healthy parents (sequencing data not shown) and the control

*OPA1* cDNA analysis in family 2 revealed two different cDNA species, including the wildtype transcript, which was present in all analyzed individuals including the control. An additional smaller transcript was reported in the index patient (Fig. 3b). The unaffected parents, who do not harbor the deletion showed only the wildtype form (Fig. 3b). Sequence analysis of the additional smaller band found in the index patient disclosed a skipping of exons 18 and 19 (Fig. 5). The heterozygous c.2012 + 4_2012 + 7delAGTA deletion in patient 1 occurred therefore *de novo* and is likely to be pathogenic, since it is absent in both healthy parents.

## Discussion

*OPA1* encodes a protein located in the inner mitochondrial membrane and is involved in a number of different cellular processes. It plays a key role during mitochondrial inner membrane fusion, further it is involved in mitochondrial DNA, respiratory chain and membrane potential maintenance, cristae organization by controlling their shape and structure and it has been shown to play a role in apoptosis [22–25]. Over 300 pathogenic mutations have been reported in *OPA1* [33] of which most mutations lead to the generation of a PTC and result in a loss of function of one allele (haploinsufficiency), which is proposed to be the major mechanism underlying the pathogenesis of ADOA [20, 28]. The OPA1 protein consists of a hydrophobic transmembrane domain, a GTPase domain, a middle domain and two C-terminal coiled-coil domain. Most of the reported mutations are located within the GTPase domain and the C-terminal coiled-coil domain (exon 27 and 28) known as mutational hotspots of *OPA1.*

Our study indicates that ADOA in two unrelated patients and their affected family members is caused by two novel splice region mutations in *OPA1*. We screened the entire coding and flanking region of *OPA1* in the two index patients and we identified two novel splice region mutations. The heterozygous c.[611-37_611-38insACTGGAGAATGTAAAGGGCTTT;611-6_611-16delCATATTTATCT] leads to the activation of a cryptic splice site resulting in intron retention and a *de novo* heterozygous 4 bp deletion c.2012 + 4_2012 + 7delAGTA leads to an in-frame exon 18 and 19 skipping. Both heterozygous splice region mutations, c.[611-37_611-38insACTGGAGAATGTAAAGGGCTTT;611-6_611-16delCATATTTATCT] in intron 5 and c.2012 + 4_2012 + 7delAGTA in intron 19 of the *OPA1* gene, have not been described before.

The novel splice region mutation c.[611-37_611-38insACTGGAGAATGTAAAGGGCTTT;611-6_611-16delCATATTTATCT] identified in family 1 is likely to cause premature termination of translation of *OPA1* by the formation of a PTC*,* leading to a truncated protein that lacks around 80% of the OPA1 protein. Furthermore, formation of a premature stop codon located within and not at the end of the transcript, more than 50-55 nucleotides upstream of the last exon-exon junction, raises the possibility that non-sense mediated decay (NMD) might be involved in degrading aberrant transcripts [32, 34]. Since we identified a mutant transcript species without inhibiting NMD, we conclude that NMD was not sufficient enough to entirely prevent the expression of truncated OPA1 and that these small amounts could interfere with both, the structural as well as the regulatory function of OPA1 in a negative way.

The *de novo* splice region mutation c.2012 + 4_2012 + 7delAGTA found in the affected child of family 2, leads to an in-frame skipping of exon 18 and 19 which affects the middle domain of the OPA1 protein. The middle domain of dynamins has been shown to be possibly involved in self- and higher-order assembly [35, 36]. Therefore, disruption of the middle domain of OPA1 may result in impaired mitochondrial localization which further lead to a decreased mitochondrial inner membrane fusion and a subsequent mitochondrial network fragmentation. Fragmentation of the mitochondrial network triggers decreased energy supply due to the loss of mitochondrial bioenergetics integrity on one hand and an increased production of ROS on the other hand. This leads finally to mitochondrial dysfunction and to cell death, initiated by the release of cytochrome c from mitochondria [24].

We suggest that the activation of a cryptic splice site and the in-frame exon skipping

might affect the OPA1 protein stability. Therefore, we reason that the mutant OPA1 protein is unstable and gets rapidly degraded, either through non-sense mediated decay or another degradation mechanism leading to haploinsufficiency. A further speculative possibility, especially for the *de novo* splice region mutation c.2012 + 4_2012 + 7delAGTA, leading to an in-frame exon skipping, could lead to an altered protein structure and function instead of resulting in haploinsufficiency, however, this would need to be elucidated by additional functional studies.

The exact pathomechanism remains unknown and therefore, further studies, preferably in vivo, would shed light on the pathogenic mechanisms playing a role during development of ADOA caused by *OPA1* mutations.

We suggest that in both analyzed families the altered protein length and structure is likely the mechanism for ADOA in the affected individuals, therefore, analysis at the genomic as well as at the RNA level should be performed to investigate *OPA1* defects.

Because of the mitochondrial dysfunction and the resulting impaired energy supply, the optic nerve could be primarily affected due to its higher sensitivity to energy deficiency leading to optic atrophy [37] in ADOA. To understand the pathophysiology of ADOA is important for the development of novel therapeutic strategies. To this day, there is no cure for ADOA, but a study performed by Barboni et al. in 2013, showed for the first time an improved visual function, after application of a synthetic analogue of coenzyme Q10 (Idebenone), an antioxidant used to reduce reactive oxygen species (ROS) levels in Leber’s hereditary optic neuropathy (LHON) patients, in ADOA patients with *OPA1* mutations [38]. Further, identification of new mutations and their associated clinical presentation would enable an appropriate genetic counseling of the patients and their family members. Our current knowledge about the exact function and the pathomechanism of *OPA1* is far from complete, but identification of new mutations associated with ADOA and their functional characterization would give us new insights into the pathogenesis of ADOA.

## Conclusion

Many of the reported *OPA1* mutations have only been characterized at the genomic level, only a few splice site or splice region variants have been investigated at their transcript level. In our study, we were able to characterize two novel splice region mutations at their genomic, as well as at their RNA level. Our results showed the importance of cDNA analysis to evaluate the effect of intronic DNA variants of unknown clinical significance encompassing the splice regions.

The study further expands the mutational spectrum of *OPA1* in ADOA by the discovery of two novel splice region mutations and their characterization at the RNA level in individuals presenting with the clinical picture of an isolated ADOA, since there was no evidence of extra-ocular features. We could further confirm the variability of intrafamilial phenotypic expression associated with *OPA1* mutations, which was shown in family 1 with its incomplete penetrance, ranging from nearly subclinical, mildly affected individuals to symptomatic family members presenting with visual impairment and optic atrophy. The clinical phenotype could be dependent on the quantity of aberrant OPA1 protein disturbing normal mitochondrial function. However, the final clinical manifestation is likely to be influenced by other endogenous and/or environmental modifying factors.

## Additional file

Additional file 1: Additional 5’ M13 Sequence: F: 5’-GTAAAACGACGGCCAGT-3’, R: 5’-CAGGAAACAGCTATGAC-3’PCR was performed according to standard PCR conditions. (DOCX 77 kb)

## Abbreviations

ADOA: Autosomal dominant optic atrophy
BCVA: Best corrected visual acuity
cDNA: Complementary DNA
gDNA: Genomic DNA
LHON: Leber’s hereditary optic neuropathy
MLPA: Multiplex ligation-dependent probe amplification
NMD: Nonsense-mediated decay
PTC: Premature termination codon
ROS: Reactive oxygen species
RT-PCR: Reverse transcription polymerase chain reaction
VOUS: Variants of unknown significance

## References

[1] Kjer P (1959). Infantile optic atrophy with dominant mode of inheritance: a clinical and genetic study of 19 Danish families. Acta Ophthalmol Suppl.

[2] Delettre C, Lenaers G, Pelloquin L, Belenguer P, Hamel CP (2002). OPA1 (Kjer type) dominant optic atrophy: a novel mitochondrial disease. Mol Genet Metab.

[3] Kivlin JD, Lovrien EW, Bishop DT, Maumenee IH (1983). Linkage analysis in dominant optic atrophy. Am J Hum Genet.

[4] Kjer B, Eiberg H, Kjer P, Rosenberg T (1996). Dominant optic atrophy mapped to chromosome 3q region. II. Clinical and epidemiological aspects. Acta Ophthalmol Scand.

[5] Amati-Bonneau P, Milea D, Bonneau D, Chevrollier A, Ferre M, Guillet V, Gueguen N, Loiseau D, de Crescenzo MA, Verny C (2009). OPA1-associated disorders: phenotypes and pathophysiology. Int J Biochem Cell Biol.

[6] Cohn AC, Toomes C, Potter C, Towns KV, Hewitt AW, Inglehearn CF, Craig JE, Mackey DA (2007). Autosomal dominant optic atrophy: penetrance and expressivity in patients with OPA1 mutations. Am J Ophthalmol.

[7] Abegg M, Zinkernagel M, Wolf S (2014). Re: Ronnback et al.: Imaging of the macula indicates early completion of structural deficit in autosomal-dominant optic atrophy (Ophthalmology 2013;120:2672-7). Ophthalmology.

[8] Votruba M, Moore AT, Bhattacharya SS (1998). Clinical features, molecular genetics, and pathophysiology of dominant optic atrophy. J Med Genet.

[9] Yu-Wai-Man P, Griffiths PG, Hudson G, Chinnery PF (2009). Inherited mitochondrial optic neuropathies. J Med Genet.

[10] Yu-Wai-Man P, Griffiths PG, Chinnery PF (2011). Mitochondrial optic neuropathies - disease mechanisms and therapeutic strategies. Prog Retin Eye Res.

[11] Kerrison JB, Arnould VJ, Ferraz Sallum JM, Vagefi MR, Barmada MM, Li Y, Zhu D, Maumenee IH (1999). Genetic heterogeneity of dominant optic atrophy, Kjer type: Identification of a second locus on chromosome 18q12.2-12.3.. Arch Ophthalmol.

[12] Reynier P, Amati-Bonneau P, Verny C, Olichon A, Simard G, Guichet A, Bonnemains C, Malecaze F, Malinge MC, Pelletier JB (2004). OPA3 gene mutations responsible for autosomal dominant optic atrophy and cataract. J Med Genet.

[13] Barbet F, Hakiki S, Orssaud C, Gerber S, Perrault I, Hanein S, Ducroq D, Dufier JL, Munnich A, Kaplan J (2005). A third locus for dominant optic atrophy on chromosome 22q. J Med Genet.

[14] Delettre C, Lenaers G, Griffoin JM, Gigarel N, Lorenzo C, Belenguer P, Pelloquin L, Grosgeorge J, Turc-Carel C, Perret E (2000). Nuclear gene OPA1, encoding a mitochondrial dynamin-related protein, is mutated in dominant optic atrophy. Nat Genet.

[15] Eiberg H, Kjer B, Kjer P, Rosenberg T (1994). Dominant optic atrophy (OPA1) mapped to chromosome 3q region. I. Linkage analysis. Hum Mol Genet.

[16] Votruba M, Moore AT, Bhattacharya SS (1998). Demonstration of a founder effect and fine mapping of dominant optic atrophy locus on 3q28-qter by linkage disequilibrium method: a study of 38 British Isles pedigrees. Hum Genet.

[17] Olichon A, Emorine LJ, Descoins E, Pelloquin L, Brichese L, Gas N, Guillou E, Delettre C, Valette A, Hamel CP (2002). The human dynamin-related protein OPA1 is anchored to the mitochondrial inner membrane facing the inter-membrane space. FEBS Lett.

[18] Votruba M, Moore AT, Bhattacharya SS (1997). Genetic refinement of dominant optic atrophy (OPA1) locus to within a 2 cM interval of chromosome 3q. J Med Genet.

[19] Jonasdottir A, Eiberg H, Kjer B, Kjer P, Rosenberg T (1997). Refinement of the dominant optic atrophy locus (OPA1) to a 1.4-cM interval on chromosome 3q28-3q29, within a 3-Mb YAC contig. Hum Genet.

[20] Delettre C, Griffoin JM, Kaplan J, Dollfus H, Lorenz B, Faivre L, Lenaers G, Belenguer P, Hamel CP (2001). Mutation spectrum and splicing variants in the OPA1 gene. Hum Genet.

[21] Alexander C, Votruba M, Pesch UE, Thiselton DL, Mayer S, Moore A, Rodriguez M, Kellner U, Leo-Kottler B, Auburger G (2000). OPA1, encoding a dynamin-related GTPase, is mutated in autosomal dominant optic atrophy linked to chromosome 3q28. Nat Genet.

[22] Belenguer P, Pellegrini L (2013). The dynamin GTPase OPA1: more than mitochondria?. Biochim Biophys Acta.

[23] Alavi MV, Fuhrmann N (2013). Dominant optic atrophy, OPA1, and mitochondrial quality control: understanding mitochondrial network dynamics. Mol Neurodegener.

[24] Olichon A, Baricault L, Gas N, Guillou E, Valette A, Belenguer P, Lenaers G (2003). Loss of OPA1 perturbates the mitochondrial inner membrane structure and integrity, leading to cytochrome c release and apoptosis. J Biol Chem.

[25] MacVicar T, Langer T (2016). OPA1 processing in cell death and disease - the long and short of it. J Cell Sci.

[26] Ferre M, Amati-Bonneau P, Tourmen Y, Malthiery Y, Reynier P (2005). eOPA1: an online database for OPA1 mutations. Hum Mutat.

[27] Votruba M, Fitzke FW, Holder GE, Carter A, Bhattacharya SS, Moore AT (1998). Clinical features in affected individuals from 21 pedigrees with dominant optic atrophy. Arch Ophthalmol.

[28] Pesch UE, Leo-Kottler B, Mayer S, Jurklies B, Kellner U, Apfelstedt-Sylla E, Zrenner E, Alexander C, Wissinger B (2001). OPA1 mutations in patients with autosomal dominant optic atrophy and evidence for semi-dominant inheritance. Hum Mol Genet.

[29] Skidd PM, Lessell S, Cestari DM (2013). Autosomal dominant hereditary optic neuropathy (ADOA): a review of the genetics and clinical manifestations of ADOA and ADOA+. Semin Ophthalmol.

[30] Carelli V, Musumeci O, Caporali L, Zanna C, La Morgia C, Del Dotto V, Porcelli AM, Rugolo M, Valentino ML, Iommarini L (2015). Syndromic parkinsonism and dementia associated with OPA1 missense mutations. Ann Neurol.

[31] Toomes C, Marchbank NJ, Mackey DA, Craig JE, Newbury-Ecob RA, Bennett CP, Vize CJ, Desai SP, Black GC, Patel N (2001). Spectrum, frequency and penetrance of OPA1 mutations in dominant optic atrophy. Hum Mol Genet.

[32] Frischmeyer PA, Dietz HC (1999). Nonsense-mediated mRNA decay in health and disease. Hum Mol Genet.

[33] Ferre M, Bonneau D, Milea D, Chevrollier A, Verny C, Dollfus H, Ayuso C, Defoort S, Vignal C, Zanlonghi X (2009). Molecular screening of 980 cases of suspected hereditary optic neuropathy with a report on 77 novel OPA1 mutations. Hum Mutat.

[34] Schweingruber C, Rufener SC, Zund D, Yamashita A, Muhlemann O (2013). Nonsense-mediated mRNA decay - mechanisms of substrate mRNA recognition and degradation in mammalian cells. Biochim Biophys Acta.

[35] Chang CR, Manlandro CM, Arnoult D, Stadler J, Posey AE, Hill RB, Blackstone C (2010). A lethal de novo mutation in the middle domain of the dynamin-related GTPase Drp1 impairs higher order assembly and mitochondrial division. J Biol Chem.

[36] Ramachandran R, Surka M, Chappie JS, Fowler DM, Foss TR, Song BD, Schmid SL (2007). The dynamin middle domain is critical for tetramerization and higher-order self-assembly. EMBO J.

[37] Ito Y, Nakamura M, Yamakoshi T, Lin J, Yatsuya H, Terasaki H (2007). Reduction of inner retinal thickness in patients with autosomal dominant optic atrophy associated with OPA1 mutations. Invest Ophthalmol Vis Sci.

[38] Barboni P, Valentino ML, La Morgia C, Carbonelli M, Savini G, De Negri A, Simonelli F, Sadun F, Caporali L, Maresca A (2013). Idebenone treatment in patients with OPA1-mutant dominant optic atrophy. Brain.

[39] Zhang MQ (1998). Statistical features of human exons and their flanking regions. Hum Mol Genet.

[40] Yeo G, Burge CB (2004). Maximum entropy modeling of short sequence motifs with applications to RNA splicing signals. J Comput Biol.

[41] Reese MG, Eeckman FH, Kulp D, Haussler D (1997). Improved splice site detection in Genie. J Comput Biol.

[42] Pertea M, Lin X, Salzberg SL (2001). GeneSplicer: a new computational method for splice site prediction. Nucleic Acids Res.

