# Supplementary material for: Characterization of two novel intronic OPA1 mutations resulting in aberrant pre-mRNA splicing
Source: BMC Med Genet. 2017 Feb 28;18:22. doi: 10.1186/s12881-017-0383-x (PMC5331656; doi:10.1186/s12881-017-0383-x)
Supplement: Supplementary file 1 [file 12881_2017_383_MOESM1_ESM.docx]

Primer	Sequences*	Annealing temp. (°C)
*OPA1*Ex1 F	5'-ACTTCCTGGGTCATTCCTGGAC-3'	60
*OPA1*Ex1 R	5'-CATCTGGGAATTCTCCAACTGC-3'	
*OPA1*Ex2 F	5'-CATTTCCTTTGTACTGTTACCC-3'	60
*OPA1*Ex2 R	5'-CATGATATAATAATTGGAAAACC-3'	
*OPA1*Ex3 F	5'-TTTTCTTTACATGTTTATTTGGC-3'	60
*OPA1*Ex3 R	5'-AATAAACTATTTCTCTTTCCTCG-3'	
*OPA1*Ex4 F	5'-AGTAGCTGTTTTGTAGGGTTGTC-3'	55
*OPA1*Ex4 R	5'-AATAATCCATGTATTTTTCCTCC-3'	
*OPA1*Ex4b F	5'-TATTTGCCCAATTATTGCCC-3'	60
*OPA1*Ex4b R	5'-AAGGATAATGAGTTTGCCTAATA-3'	
*OPA1*Ex5 F	5'-TAGGCTGTTGACATCACTGGA-3'	55
*OPA1*Ex5 R	5'-TATTTGCTTCTCAGATGTTATCA-3'	
*OPA1*Ex5b F	5'-ACCATCCCTCCCTAGCTTAC-3'	60
*OPA1*Ex5b R	5'-CCATGAACAGATTGAGGTGA-3'	
*OPA1*Ex6 F	5'-ACTTGCTGTACATTCTGTATAA-3'	55
*OPA1*Ex6 R	5'-TGTCCATATCACCAAAGTTA-3'	
*OPA1*Ex7 F	5'-GACTTAATACTATTTGATAACCC-3'	55
*OPA1*Ex7 R	5'-GACAGAATACTTTACAGCTCC-3'	
*OPA1*Ex8 F	5'-AAGATGTATTTATGTAATTTGAG-3'	55
*OPA1*Ex8 R	5'-TACATCTAGTATGTATATATGTG-3'	
*OPA1*Ex9 F	5'-AAAACTCAGAGCAGCATTACAAA-3'	60
*OPA1*Ex9 R	5'-TGAAGCAGTTACTCTCCTAAGG-3'	
*OPA1*Ex10_11 F	5'-GGCTGTGGGAATTAATATTAC-3'	60
*OPA1*Ex10_11 R	5'-GCAATCTAAACATCAATACCTA-3'	
*OPA1*Ex12_13 F	5'-AATATATCATTTTGTGGGTAAT-3'	55
*OPA1*Ex12_13 R	5'-TGCATTTATCTTGTATGTTTAC-3'	
*OPA1*Ex14 F	5'-GGGGTATAATTTGTACTGAGTT-3'	55
*OPA1*Ex14 R	5'-TTTAAATAACAAATCCCTATCA-3'	
*OPA1*Ex15_16 F	5'-ACGCTTTTAAAACTTATGTAAAC-3'	60
*OPA1*Ex15_16 R	5'-ACAGTTCAATTTAAGCTACTCTC-3'	
*OPA1*Ex17 F	5'-GAGTGGCTGTTAGCAAGCAC-3'	60
*OPA1*Ex17 R	5'-AGAATCGTATGGATGCCAAAGA-3'	
*OPA1*Ex18 F	5'-ACATCTGGAAAGAAGGAGGG-3'	58
*OPA1*Ex18 R	5'-CTGCTCCTAGAGATGTATTC-3'	
*OPA1*Ex19 F	5'-CTCTGAAAATCATGACAGGGT-3'	60
*OPA1*Ex19 R	5'-CAAGGCAACAATAAATCACTG-3'	
*OPA1*Ex20 F	5'-GTTAATGATACTTCAGTCAAGC-3'	55
*OPA1*Ex20 R	5'-TAAAATTCACAGCTCCTACTC-3'	
*OPA1*Ex21 F	5'-CACATCTGTTTGGCTTGAGC-3'	55
*OPA1*Ex21 R	5'-TGAAAAAGTATCAATTTGAGAAG-3'	
*OPA1*Ex22 F	5'-TATTTACTAAGCTGTCAATTTG-3'	55
*OPA1*Ex22 R	5'-AATGTTCACTAAAGTTGGATG-3'	
*OPA1*Ex23 F	5'-TGAAATAGTTAAGAAAGCAAGA-3'	55
*OPA1*Ex23 R	5'-AGAGCCACAAAAAGGTTATA-3'	
*OPA1*Ex24 F	5'-CACCAAATTATGAACCATCT-3'	55
*OPA1*Ex24 R	5'-GACAAAAGTCAAATTAAGCACT-3'	
*OPA1*Ex25 F	5'-TGTACAACTTCTCAGTGTGG-3'	55
*OPA1*Ex25 R	5'-AAGGACTTAAAATTAATGCTTA-3'	
*OPA1*Ex26 F	5'-AGTTGTATGTGTTTACGATGAT-3'	55
*OPA1*Ex26 R	5'-TACTTTTGGGAAGTATTTTG-3'	
*OPA1*Ex27 F	5'-GGTTAATTTTATCATCTTTATTC-3'	55
*OPA1*Ex27 R	5'-TTTGAGATTAAATGGATCAAC-3'	
*OPA1*Ex28 F	5'-CGCAAATAGTTAAGTATACCA-3'	58
*OPA1*Ex28 R	5'-CATTGAAATTACAGCATAAG-3'	

* Additional 5' M13 Sequence : F: 5'-GTAAAACGACGGCCAGT-3', R: 5'-CAGGAAACAGCTATGAC-3'

PCR was performed according to standard PCR conditions
